# Exercise and Nutrition in the Mental Health of the Older Adult Population: A Randomized Controlled Clinical Trial

**DOI:** 10.3390/nu16111741

**Published:** 2024-06-01

**Authors:** María del Carmen Carcelén-Fraile, Noelia del Pino Déniz-Ramírez, Jessica Sabina-Campos, Agustín Aibar-Almazán, Yulieth Rivas-Campo, Ana María González-Martín, Yolanda Castellote-Caballero

**Affiliations:** 1 Department of Education and Psychology, Faculty of Social Sciences, University of Atlántico Medio, 35017 Las Palmas de Gran Canaria, Spain; 2 Vecindario Rehabilitation Center, 35110 Las Palmas de Gran Canaria, Spain; 3 Santa Cruz Rehabilitation Center, 38005 Santa Cruz de Tenerife, Spain; 4 Department of Health Sciences, Faculty of Health Sciences, University of Jaén, 23071 Jaén, Spain; 5 Department of Health Sciences, Faculty of Health Sciences, University of Atlántico Medio, 35017 Las Palmas de Gran Canaria, Spain; 6 Faculty of Human and Social Sciences, University of San Buenaventura-Cali, Santiago de Cali 760016, Colombia; 7 Department of Psychology, Higher Education Center for Teaching and Educational Research, Plaza de San Martín, 4, 28013 Madrid, Spain

**Keywords:** resistance program, Mediterranean diet, anxiety, depression, sleep quality, perceived stress

## Abstract

(1) Background: Global population aging is changing demographic structures and presents significant challenges for health systems, which must adapt to an increasingly elderly population. (2) Methods: The study employed a randomized controlled trial design with a total of 116 older adults aged 65 or older, randomly assigned to an experimental group (*n* = 57) undergoing a combined resistance program and Mediterranean diet program and a control group (*n* = 59) who did not receive any intervention. Anxiety and depression were evaluated using the Hospital Anxiety and Depression Scale (HADS), sleep quality through the Pittsburgh Sleep Quality Index (PSQI), and perceived stress using the Perceived Stress Scale (PSS). (3) Results: Statistically significant improvements (*p* < 0.05) were observed both within and between groups in anxiety (Cohen’s d = 0.38 and 0.78, respectively), depression (Cohen’s d = 0.56 and 0.18, respectively), perceived stress (Cohen’s d = 0.15 and 0.39, respectively), and in the PSQI domains: subjective sleep quality (Cohen’s d = 1.01 and 0.53, respectively), sleep duration (Cohen’s d = 0.112 and 0.53, respectively), sleep disturbances (Cohen’s d = 1.92 and 0.95, respectively), use of medications (Cohen’s d = 0.34 and 0.40, respectively), and the PSQI total score (Cohen’s d = 0.68 and 0.49, respectively). No significant differences were observed in sleep latency or daytime dysfunction. (4) Conclusions: The results of the present study suggest that resistance intervention may be an effective therapeutic option to improve mental health and sleep quality in older adults aged 65 or older, offering a non-pharmacological approach to improving overall well-being in this demographic.

## 1. Introduction

Demographic change and the consequent aging of the population worldwide are highly relevant phenomena that are transforming the population structure throughout the world [1]. In recent decades, we have witnessed a significant increase in the proportion of people over 65 years of age in relation to the total population [2]. In Spain, population aging is a phenomenon that has been increasing in recent decades. According to data from the National Institute of Statistics (INE), in 2020, approximately 20.5% of the Spanish population was 65 years old or older, and this percentage is expected to continue increasing in the coming years [3]. This demographic phenomenon is due to a combination of factors, including declining birth rates and increasing life expectancy, driven by medical advances and improvements in living conditions [4]. The current demographic transition presents significant challenges for health systems in adapting to an aging and diverse population. Specialized care services are needed to address the specific needs of older adults, including home, long-term, and mental health care [5]. Improving coordination and integration of health services is crucial to ensuring continued quality care for this demographic. In addition, it is important to implement prevention and health promotion strategies with a focus on social determinants specifically aimed at older adults to promote their general well-being [6]. The aging process has a series of significant implications in terms of mental health, burden of disease, and health services [7]. First, aging has been found to be associated with an increased risk of mental disorders, such as depression and anxiety, as well as a higher prevalence of neurodegenerative diseases, such as Alzheimer’s disease and other dementias [8]. This represents a significant challenge for health systems, requiring increased attention and resources to address these conditions effectively [9]. According to data provided by the World Health Organization (WHO), it is estimated that approximately 15% of individuals aged 60 or older have some type of mental disorder. Among these disorders, depression and anxiety stand out as the most prevalent in this demographic [10]. Specifically, it is estimated that around 7% of people over 65 years of age suffer from depression worldwide, this being one of the main mental health concerns for this age group [11]. Regarding anxiety, studies indicate that between 10% and 20% of older adults experience some type of anxiety disorder [12]. Furthermore, sleep disorders are common in this population segment, with approximately half of older adults affected, which leads to difficulties falling or staying asleep [13]. On the other hand, perceived stress, generated by concerns related to health, economic situation, social isolation, and other factors, can also affect the mental health of older adults. Although specific numbers are more difficult to determine due to their subjective nature, perceived stress is recognized as an additional concern in this demographic. In this context, the search for effective and comprehensive interventions becomes crucial to improving the quality of life of this vulnerable population; therefore, the role of nutrition in the aging process, especially in the context of older adults, becomes vitally important [14]. During the aging process, the body experiences a series of changes, such as decreased muscle mass, decreased bone density, and reduced immune system function [15]. These changes can increase susceptibility to various diseases and health conditions, making a proper diet even more crucial [16]. The relationship between diet and mental health is the object of study in scientific research, revealing a significant connection between food intake and psychological well-being [17]. The essential nutrients present in a balanced diet play a crucial role in regulating brain and central nervous system function [18]. For example, omega-3 fatty acids, abundant in foods such as fish, nuts, and seeds, have been shown to positively influence mental health while reducing the risk of mood disorders such as depression and anxiety [19].

On the contrary, an unhealthy diet, characterized by high consumption of refined sugars, saturated fats, and processed foods, has been associated with an increased risk of mood disorders and mental health problems. These foods can induce fluctuations in blood glucose levels and systemic inflammation, both factors that affect brain function and mood. Additionally, unhealthy dietary patterns have been correlated with an increased risk of obesity and chronic diseases, such as type 2 diabetes and cardiovascular disease [20], which, in turn, are related to an increase in mental health disorders such as mood and mental health problems such as depression [21].

The Mediterranean diet has emerged as an especially beneficial dietary option for older adults. Based on the traditional dietary patterns of Mediterranean regions, this diet is characterized by a high consumption of fruits, vegetables, legumes, nuts, fish, and olive oil, along with a moderate intake of dairy, poultry, and eggs, and a low consumption of red meat and processed products. This diet not only provides essential nutrients for health but also promotes a healthy lifestyle in general, encouraging regular physical activity and the enjoyment of meals in company [22]. This is particularly relevant for older adults, as it can contribute to improving their physical, emotional, and social well-being and maintaining a good quality of life as they age [23].

For its part, physical exercise is essential to promoting good health at all ages. On a mental level, it highlights the improvement in mood, the reduction of stress and anxiety, as well as the improvement in the quality of sleep [24]. Additionally, exercise prevents chronic diseases and has long-term effects on physical and mental well-being [25]. Currently, a variety of exercises are being performed with diverse objectives. Resistance training (RT) stands out as a form of strength training exercise that focuses on gradually increasing the load on muscles to develop strength. In this type of training, muscles are required to exert force against an external load, and it can be safe and effective for older adults [26]. Additionally, several studies have supported the benefits of resistance training for the physical and mental health of older people [27,28,29]. Scientific research has shown that the regular practice of resistance training can significantly contribute to the prevention and management of common chronic diseases in this population, such as cardiovascular disease and type 2 diabetes [30], because it has the potential to improve sensitivity to insulin and improve glucose oxidation [31]. Additionally, resistance training has been found to help improve body composition by reducing body fat and increasing lean muscle mass, which may have positive effects on metabolism and overall physical function [32]. On the other hand, resistance training has been associated with significant improvements in the quality of life and psychological well-being of older people [33]. Studies have found that regular participation in resistance training programs can reduce symptoms of depression and anxiety, as well as improve self-esteem and perceptions of emotional well-being in older adults [34,35,36]. Furthermore, it has been observed that resistance training can promote autonomy and functional independence in activities of daily living, contributing to a greater sense of satisfaction and fulfillment in life for older people [37].

This research aims to shed light on the effectiveness of resistance programs and the Mediterranean diet in managing anxiety, depression, sleep, and stress in this specific demographic group.

## 2. Materials and Methods

### 2.1. Study Design

A randomized, controlled study was designed to evaluate the effectiveness of a 12-week combined resistance and diet program on anxiety, depression, sleep quality, and stress in older adults aged 65 or older. The study was registered prior to its initiation in “ClinicalTrials.gov” with the reference approval regis NCT06426589 and received approval from the Ethics Committee of the Mid-Atlantic University (CEI/02-12).

### 2.2. Participants

**Figure 1:**
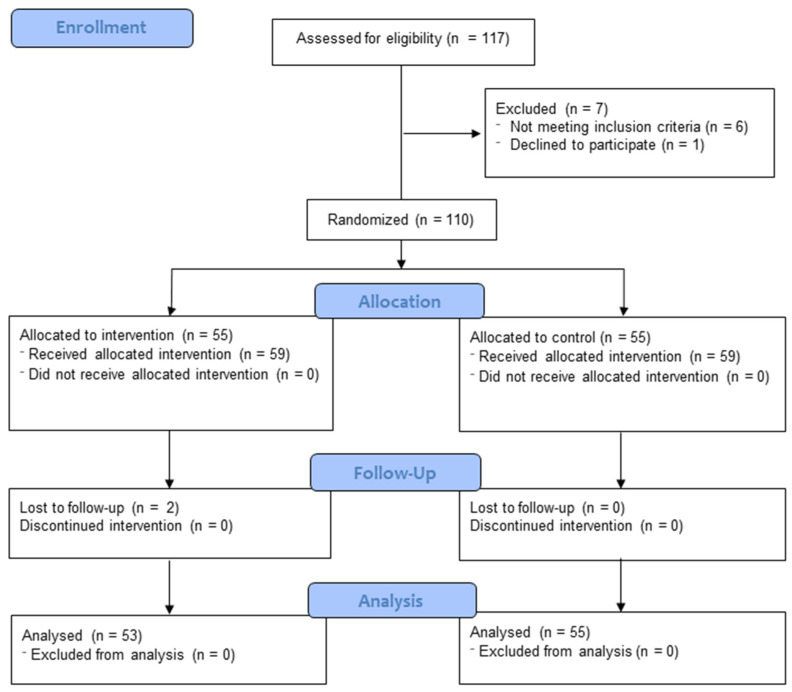
Flow diagram of the study participants.

At the beginning of the study, a total of 117 older adults aged 65 or older were contacted, of whom 118 were eligible to participate and, therefore, were integrated into the study (Figure 1). The search for participants was carried out from October to November of 2023 through the use of telephone calls and emails. Those interested in taking part in the study signed an informed consent form in accordance with the Declaration of Helsinki, the Standards of Good Clinical Practice, and all relevant regulations and laws. This form detailed the objectives, procedures, possible risks and benefits, and the confidentiality of the information collected. Older adults aged 65 or older who had the following pre-requisites: (i) were 65 years of age or older; (ii) had not been part of any resistance program in the last 12 months; and (iii) could understand and follow the instructions, activities, and protocols of the exercise program. Participants were excluded if they had the following: (i) they suffered from any systemic condition (e.g., neurodegenerative, musculoskeletal, or visual diseases) that prevented them from performing the exercises; (ii) they had any vestibular disease or disorder; (iii) they consumed medications that influenced the central nervous system, balance, or coordination (for example, antidepressants, vestibular sedatives, or anxiolytics).

### 2.3. Randomization

The allocation of participants was carried out by using a computer-generated series of random numbers, equally distributing subjects between the experimental group (EG) and the control group (CG) in a 1:1 ratio. The distribution of groups remained confidential, unknown to both the participants, the researchers, and the physical therapist in charge of implementing the intervention. For this purpose, opaque envelopes were used, sealed, and numbered in order, which were stored under lock and key and could only be opened by someone outside the study. Once this process was completed, 55 subjects were assigned to the experimental group and 55 to the control group.

### 2.4. Intervention

#### Resistance Training

Subjects assigned to the experimental group participated in a resistance program twice a week (on Tuesdays and Thursdays) for a period of 12 weeks, totaling 24 sessions of 60 min each. Each session during this time was divided into three clearly differentiated parts: (i) A 5-min warm-up comprised of a series of gentle, low-intensity exercises designed to gradually prepare the muscles and joints of older adults for the main exercise. This portion included flexibility and stretching movements, as well as joint mobility exercises; (ii) 50 min dedicated to the main section of the intervention; and (iii) 5 min for relaxation techniques that incorporate flexibility exercises and stretching. In the main section, various resistance movements were performed: leg extension and leg curl machine, barbell bench press, lateral pulldown, barbell curl, overhead press, and triceps extension machine. Resistance movements were performed in three sets with 10 repetitions and an intensity of 75% of one repetition maximum (1 RM). Rest intervals between sets and between movements were 2 and 3 min, respectively.

Each session was led by a qualified and experienced professional. There were no incidents of injuries or negative effects reported during the duration of the intervention. To be considered in the final analysis, participants had to attend at least 80% of the stipulated sessions. Members of the control group maintained their daily activities, received guidance to encourage physical activity, and were prevented from engaging in any type of formal training program. Throughout the 12 weeks of intervention, they were followed up regularly by telephone to inquire about their physical activity habits.

Mediterranean Diet. In addition to the resistance intervention, the experimental group received a Mediterranean diet protocol based on the study by Ismail et al. [38] with the following meal plan: (i) carbohydrates constituted 50% of daily intake; (ii) fats represented 35%; and (iii) proteins accounted for 15%. Considering this plan, weekly consumption recommendations were: (i) the use of 320 mL of extra virgin olive oil was recommended; (ii) an intake of 30 g of cereals or bread was advised; (iii) the combination of whole grains, fruits, and vegetables should be 125 mL; (iv) consuming 100 g of eggs daily was suggested; (v) legumes and nuts should be consumed in quantities of 175 mL and 30 g, respectively; (vi) red meat was limited to a weekly portion of 75 g; (vii) a daily consumption of 75 g of fish and poultry was indicated; and (viii) low-fat dairy products were recommended, equivalent to 250 mL of milk, 50 g of cheese, and 175 g of yogurt. Additionally, reducing intake of certain products, such as processed meats, cream, butter, sugary drinks, cookies, bread, and other refined cereals, was encouraged. To ensure compliance with the diet, each participant had a food diary to record their daily intake. The 14-item MEDAS questionnaire measured adherence to the Mediterranean diet. Additionally, based on participants’ dietary preferences, intolerances, or allergies, individual adjustments were made within the framework of the Mediterranean diet to ensure compliance without compromising basic dietary principles.

The members of the control group continued their daily activities unchanged, receiving guidance to promote physical activity while limiting their participation in any type of organized training program, and they were advised to maintain their usual dietary habits. Throughout the 12-week intervention, regular follow-ups were conducted via phone calls to inquire about their physical activity habits.

### 2.5. Outcomes

All data were collected at the beginning and at the end of the intervention period. Sociodemographic characteristics such as age, sex, occupational status, marital status, and educational level were recorded before assignment in the presence of experienced interviewers.

#### 2.5.1. Adherence to the Mediterranean Diet

This was measured using the 14-item MEDAS questionnaire, developed by the PREDIMED researchers. The questionnaire consists of 12 queries regarding how often various foods are eaten and two additional questions concerning typical eating habits in Spain [39]. Each question could be answered with a score of either zero or one. A score of one was given for using olive oil as the primary cooking fat, choosing white over red meat, and consuming at least four tablespoons (one tablespoon = 13.5 g) of olive oil daily (used in cooking, as a salad dressing, etc.), two or more vegetable servings per day, three or more fruit servings daily, fewer than one serving per day of red meat or processed meats, less than one serving of animal fats daily, and under one cup (one cup = 100 mL) of sweetened or fizzy beverages daily. Points were also awarded for drinking at least seven glasses of wine per week, eating three or more servings of legumes weekly, three or more servings of fish, no more than two industrial bakery items per week, three or more servings of nuts, and at least two servings of sofrito each week (a sauce prepared with tomato, garlic, onion, or leeks and sautéed in olive oil). The total possible score ranged from 0 to 14, with a total of 9 or higher signifying sufficient compliance with the Mediterranean diet.

#### 2.5.2. Anxiety and Depression

To evaluate anxiety and depression levels, the Hospital Anxiety and Depression Scale (HADS) was used [40,41]. This instrument is frequently used in populations of older adults who are not institutionalized [42] and includes 14 questions, divided equally between anxiety (even questions) and depression (even questions). Items are scored on a scale of 0 to 3, resulting in a total score range of 0 to 21 for both anxiety and depression, with a higher number indicating a greater presence of symptoms.

#### 2.5.3. Sleep Quality

The Pittsburgh Sleep Quality Index (PSQI) [43,44], a widely recognized tool for the assessment of sleep quality, was the questionnaire selected for this task. The PSQI includes 19 self-report items and 5 additional items that must be completed by the participant’s bed partner or roommate (the latter are used only to obtain clinical data). From these items, a global score is calculated, and the following seven dimensions or areas are evaluated: sleep quality; time to fall asleep; sleep duration; sleep efficiency; problems during sleep; consumption of sleep medication; and daytime functioning problems. The total score on the PSQI ranges from 0 to 21, with higher scores indicating poor sleep quality.

#### 2.5.4. Perceived Stress

The measurement of perceived stress was carried out using the Perceived Stress Scale (PSS) [45] in its Spanish version [46], an instrument composed of 14 questions that determines the degree of stress felt in the last month. This questionnaire uses a response system based on a five-point scale (0 = never, 1 = almost never, 2 = occasionally, 3 = frequently, and 4 = very frequently). To calculate the total score on the PSS, the response values for items 4, 5, 6, 7, 9, 10, and 13 are reversed (so that 0 becomes 4, 1 becomes 3, etc.), and then all the items are added. The score ranges from 0–56, with a higher score on the scale indicating a higher level of perceived stress.

### 2.6. Sample Size Calculation

For this study, the sample size calculation was carried out considering a mean difference of 1.27 units in the expected depression variable based on the results of Noradechanunt et al. [47]. For the calculation, a significance level of 5% and statistical power of 90% were established, which resulted in a sample size of 51 participants per group. Additionally, a dropout percentage of 15% was considered, leaving a sample of 59 participants per group for a total of 118 participants.

### 2.7. Statistical Analysis

The means and standard deviations were estimated for each of the variables under study. The difference between groups was analyzed using the Student’s *t* test for independent samples. To evaluate the effects of the intervention, a mixed analysis of variance was applied, considering the intervention (EG vs. CG) as the between-group factor and the assessment time (before treatment vs. after treatment) as the within-subject variable. The dependent variables were anxiety, depression, perceived stress, and sleep quality. Specific evaluations were carried out for each of the dependent variables. The possible interaction between the treatment (group) and the evaluation period was investigated. To determine the effect size between groups, Cohen’s d was used, interpreting values of ≤0.2 as a small effect, 0.5 as a moderate effect, and 0.8 as a large effect [48]. A *p* value less than 0.05 was accepted as statistically significant. Data analysis was performed using SPSS software, version 17.0 (SPSS, Inc., Chicago, IL, USA).

## 3. Results

The present study has 36.96% men and 63.04% women. Subjects participated in at least 91.6% of the sessions scheduled for the intervention, and no incidents of injuries or negative reactions were reported during the intervention period (Table 1).

### 3.1. Adherence to the Mediterranean Diet

According to our findings, statistically significant differences were found in nutritional status between pre- and post-measurements in the resistance training group: t (52) = −21.003, *p* = 0.000, Cohen’s d = 2.18. Moreover, statistically significant differences were found between both groups in the post-intervention measurement: t (106) = 11.729, *p* = 0.000, Cohen’s d = 1.88 (Table 2).

### 3.2. Anxiety and Depression

According to our findings, in anxiety, statistically significant differences were found between the pre- and post-measurements in the experimental group: t (52) = 3.072, *p* = 0.003, Cohen’s d = 0.38, and statistically significant differences between both groups in the post-intervention measure: t (106) = 4.212, *p* = 0.000, Cohen’s d = 0.78. Regarding depression, statistically significant differences were observed between the pre- and post-measurements in the EG: t (52) = 5.989, *p* = 0.000, Cohen’s d = 0.56. Furthermore, statistically significant differences were demonstrated between both groups in the post-intervention measure: t (106) = 2.633, *p* = 0.010, Cohen’s d = 0.18 (Table 2).

### 3.3. Perceived Stress

In perceived stress, statistically significant differences were observed between the pre- and post-measurements in the experimental group: t (52) = 2.306, *p* = 0.025, Cohen’s d = 0.15, and statistically significant differences between both groups in the post-intervention measure: t (106) = 2.098, *p* = 0.038, Cohen’s d = 0.39 (Table 2).

### 3.4. Sleep Quality

Regarding sleep quality, significant differences were found in the subdomains of: (a) subjective sleep quality; significant differences were found within the EG: t (53) = 8.638, *p* = 0.000, Cohen’s d = 1.01. Furthermore, statistically significant differences were observed between both groups in the post-intervention measure: t (106) = 2.815, *p* = 0.006, Cohen’s d = 0.53; (b) sleep duration, statistically significant differences could be observed between pre- and post-measurements in the experimental group: t (53) = 7.199, *p* = 0.000, Cohen’s d = 1.12. Likewise, statistically significant differences were observed between both groups in the post-intervention measure: t (106) = 2.888, *p* = 0.005, Cohen’s d = 0.53; (c) sleep disturbances, statistically significant differences were shown between the pre and post measurements in the EG: t (53) = 12.152, *p* = 0.000, Cohen’s d = 1.92, and statistically significant differences between both groups in the post-intervention measurement: t (106) = 25.137, *p* = 0.000, Cohen’s d = 0.95; (d) use of medications, statistically significant differences were shown between the pre- and post-measurements in the EG: t (53) = −2.020, *p* = 0.048, Cohen’s d = 0.34, and statistically significant differences between both groups in the post-intervention measurement: t (106) = −2.153, *p* = 0.033, Cohen’s d = 0.40; (e) PSQI total score, significant differences were found within the EG: t (53) = 6.741, *p* = 0.000, Cohen’s d = 0.68. Furthermore, statistically significant differences were observed between both groups in the post-intervention measure: t (106) = 2.629, *p* = 0.010, Cohen’s d = 0.49 (Table 2).

## 4. Discussion

This randomized controlled clinical trial was designed to analyze the effects of a resistance intervention combined with a Mediterranean diet for 12 weeks on anxiety, depression, sleep, and stress in institutionalized older adults aged 65 or older. The main findings of this study demonstrate significant changes pre- and post-treatments, as well as differences in favor of a resistance program and diet to reduce depression, anxiety, and perceived stress. 

The beneficial effects of resistance exercise and the Mediterranean diet on mental health and general well-being can be attributed to a series of complex mechanisms of action involving physiological, neurobiological, and psychological changes [49,50]. First, resistance exercises and the Mediterranean diet promote stress reduction by regulating the autonomic nervous system and decreasing levels of cortisol, a hormone associated with stress [51,52]. Furthermore, a recent systematic review and meta-analysis [53] found that RT is an effective non-pharmacological strategy to improve mental health outcomes in adults, regardless of their health status (with or without mental disorders). In addition, it has been shown that both resistance exercises and the Mediterranean diet have anti-inflammatory properties, which could positively contribute to mental health by reducing inflammation associated with mood disorders such as depression [54,55]. However, it is important to highlight that most previous studies have addressed these interventions independently, which highlights the originality of this study in combining both interventions. Numerous studies have demonstrated the benefits of the Mediterranean diet on the health and well-being of older adults [56,57]. This diet is associated with a reduced risk of chronic diseases, such as cardiovascular disease, type 2 diabetes, and cognitive decline [58]. Furthermore, it has been observed that adherence to the Mediterranean diet is related to a better quality of life [57], greater longevity, and a lower risk of functional disability in older adults [59]. 

At a neurobiological level, resistance exercises can influence brain plasticity and cognitive function by stimulating the release of neurotrophic factors, such as brain-derived neurotrophic factor (BDNF), that promote the growth and survival of nerve cells [60]. Additionally, resistance exercise practice has been associated with changes in brain structure and function, including a reduced volume of the amygdala, a brain region involved in the stress response [61]. Regarding the Mediterranean diet, its nutritional components can influence neurotransmission and brain function [62]. For example, omega-3 fatty acids present in fatty fish can improve communication between brain cells and reduce inflammation [63]. Similarly, polyphenols present in fruits, vegetables, and olive oil can protect against oxidative stress and improve cognitive function [64].

Depression in older people is a common and serious mental health condition that can significantly affect their quality of life and overall well-being. Therefore, it is essential to carry out strategies that promote its improvement. In a recent systematic review [65], we evaluated the strength and validity of the available observational and trial evidence on the association between a variety of dietary patterns and depression, concluding that greater adherence to the Mediterranean diet was associated with a lower risk of depression. In the present study, it has been proven that both resistance exercises and the Mediterranean diet promote mindfulness and the mind-body connection, which can improve emotional regulation and resilience to stress. Similarly, the study by Lincoln et al. [66] showed that participants who had carried out resistance exercise training obtained better scores on the geriatric depression scale, but unlike our study, they performed it on older people with type 2 diabetes over a period of 16 weeks. On the other hand, a balanced Mediterranean diet rich in fresh foods can provide essential nutrients that support mental health and general well-being [67]. 

The aging process is associated with a reduction in several sleep parameters, including total sleep time (TST), the duration of deeper sleep stages, and sleep quality [68]. In the present study, significant differences are revealed in several subdomains evaluated: subjective sleep quality, sleep duration, sleep discomfort, and medication use; Likewise, in the total score of the Pittsburgh Sleep Quality Index (PSQI), significant changes were evident in the resistance exercises and diet treatment group, which confirms the improvement in the general quality of sleep of the participants post-intervention. In line with our study, Sa Souza et al. [69] found significant improvements in different sleep parameters measured through nocturnal polysomnography after resistance exercises, but in sarcopenic older adults. Like the study by Corrêa et al. [70], in which they verified the effects of resistance training on sleep quality, but in patients on maintenance hemodialysis. For their part, the components inherent in the Mediterranean diet exert a favorable influence on sleep quality through various physiological and metabolic mechanisms [71]. Firstly, foods such as fish, dairy products, and nuts present in this diet contain tryptophan, a direct precursor of both serotonin and melatonin, essential neurotransmitters in the regulation of the circadian cycle and the induction of sleep [72]. Furthermore, the low intake of caffeine and refined sugars, characteristic of the Mediterranean diet, minimizes the adverse effects that these compounds can have on the quality and duration of sleep, thus avoiding difficulties in falling asleep and nighttime awakenings [73].

The abundant presence of antioxidants in foods such as fruits, vegetables, olive oil, and nuts contributes to reducing inflammation and oxidative stress, biological processes associated with the regulation of sleep and wakefulness [74]. Likewise, the low glycemic index of most Mediterranean foods helps maintain stable blood glucose levels, avoiding fluctuations that could interfere with the quality of sleep [75]. Finally, the relationship between the Mediterranean diet and a healthy body weight, derived from its balanced nutritional composition, constitutes a protective factor against sleep disorders such as obstructive sleep apnea and insomnia, conditions whose prevalence can be reduced with an adequate and balanced diet [76]. However, the need for additional research is highlighted to fully understand the underlying mechanisms and their applicability in various populations.

One of the highlights of the present study was the rigorous methodological design used, which was characterized by being a randomized, controlled, and double-blind clinical trial. Furthermore, a high rate of compliance by participants with the prescribed interventions was evident, and there was a considerable sample size, which increases the robustness of the findings. First, the effects of resistance training and diet will be evaluated only in the short term. Secondly, it was carried out only with Spanish-resident adults; for this reason, the findings cannot be generalized to other populations. The following implications of the results of this study suggest several important directions for future research: (i) it is essential to further explore the mechanisms underlying the benefits of resistance exercise on the mental health of older adults; (ii) the effectiveness of resistance exercises can vary significantly between different cultural and socioeconomic contexts. Therefore, it is essential to conduct studies that evaluate how factors such as diet, local customs, and access to health resources affect the results of resistance exercise programs; (iii) the long-term effects of resistance exercise also deserve attention. Although this study provides evidence of short-term benefits, future research should focus on evaluating whether these benefits are maintained, increase, or decrease over time; (iv) to effectively integrate resistance exercises into mental health care programs aimed at older adults, it is crucial to train health professionals in techniques adapted to this population; (v) establishing accessible and culturally sensitive resilience programs in healthcare settings is another key recommendation taking into account the physical limitations and cultural preferences of older adults.

## 5. Conclusions

The study carried out demonstrated that a 12-week resistance program is effective in improving various psychological and health parameters in older adults aged 65 or older. Significant improvements were observed in levels of anxiety, depression, and perceived stress, as well as in several aspects of sleep quality. in the group that participated in the resistance program compared to the control group. The significant improvements in these parameters suggest that a resistance program is a valuable intervention to promote mental health and well-being in this population, offering a viable non-pharmacological therapeutic option.

## Figures and Tables

**Table 1:** Baseline characteristics of study participants.

		Total (*n* = 108)	Experimental (*n* = 53)	Control (*n* = 55)	*p*-Value
Age. Mean (SD).		70.08 ± 2.63	69.73 ± 2.56	70.45 ± 2.68	0.460
Sex, *n* (%)	Male	33 (30.6)	15 (45.5)	18 (54.5)	0.324
	Female	75 (69.4)	38 (50.7)	37 (49.3)	
Occupational status	Retired	71 (65.7)	33 (46.5)	38 (53.5)	0.541
	Worker	2 (1.9)	2 (100.0)	0 (0.00)	
	Unemployed	35 (32.4)	18 (51.4)	17 (48.6)	
Maritial status	Single	29 (26.9)	17 (58.6)	12 (41.4)	0.686
	Married	49 (45.4)	25 (51.0)	24 (49.0)	
	Divorced/Widowed	30 (27.8)	11 (36.7)	19 (63.3)	
Educational status	Nothing	23 (21.3)	14 (60.9)	9 (39.1)	0.834
	Primary	32 (29.6)	17 (53.1)	15 (46.9)	
	Secondary	31 (28.7)	13 (41.9)	18 (58.1)	
	University	22 (20.4)	9 (40.9)	13 (59.1)	
Adherence to the Mediterranean Diet_MEDAS		6.87 ± 1.02	6.98 ± 1.01	6.76 ± 1.02	0.728
Anxiety_HADS		7.87 ± 3.31	7.49 ± 3.13	8.24 ± 3.46	0.666
Depression_HADS		9.01 ± 2.55	9.25 ± 2.67	8.78 ± 2.42	0.437
Perceived stress (PSS)		24.53 ± 13.86	23.60 ± 13.72	25.44 ± 14.06	0.751
Subjective quality_PSQI		1.72 ± 0.92	1.93 ± 0.88	1.51 ± 0.92	0.197
Latency_PSQI		1.30 ± 0.92	1.18 ± 0.89	1.42 ± 0.93	0.640
Duration_PSQI		1.85 ± 0.95	2.07 ± 0.86	1.64 ± 0.10	0.083
Efficiency_PSQI		1.53 ± 1.04	1.58 ± 1.051	1.47 ± 1.040	0.941
Discomfort_PSQI		2.27 ± 0.69	2.35 ± 1.551	2.19 ± 0.798	0.057
Medication use_PSQI		1.75 ± 0.86	1.67 ± 0.764	1.83 ± 0.950	0.076
Dysfunctions during the day_PSQI		0.71 ± 0.73	0.72 ± 0.701	0.69 ± 0.771	0.488
Total score_PSQI		10.41 ± 2.95	10.77 ± 2.982	10.07 ± 2.90	0.747

Quantitative variables are presented as the mean and standard deviation. Qualitative variables are presented as frequency and percentage. HADS: Hospital Anxiety and Depression Scale; PSQI: Pittsburgh Sleep Quality Scale; PSS: Perceived Stress Scale; SD: Standard Deviation.

**Table 2:** Effects of the resistance program on anxiety, depression, perceived stress, and sleep quality.

	Pre-Intervention		Post-Intervention		Group			Time			Group × Time		
	EG	CG	EG	CG	F (1.06)	*p* Value	η^2^	F (1.106)	*p* Value	η^2^	F (1.106)	*p* Value	η^2^
Adherence to the Mediterranean diet_MEDAS	6.98 ± 1.01	6.76 ± 1.02	9.16 ± 1.29	6.41 ± 1.23	72.710	0.000	0.389	107.084	0.000	0.484	55.325	0.000	0.327
Anxiety_HADS	7.49 ± 3.13	8.24 ± 3.46	6.26 ± 3.25	8.95 ± 3.60	11.842	0.001	0.094	0.466	0.496	0.004	6.580	0.012	0.055
Depression_HADS	7.81 ± 2.50	9.02 ± 2.45	9.25 ± 2.67	8.78 ± 2.42	0.797	0.374	0.007	8.213	0.005	0.067	15.985	0.000	0.123
Perceived stress (PSS)	23.60 ± 13.72	25.44 ± 14.06	21.47 ± 13.83	26.93 ± 14.19	2.059	0.154	0.018	0.417	0.520	0.004	13.668	0.000	0.107
Subjective quality_PSQI	1.93 ± 0.88	1.51 ± 0.92	1.14 ± 0.67	1.58 ± 0.97	0.002	0.962	0.000	43.908	0.000	0.278	61.958	0.000	0.352
Latency_PSQI	1.18 ± 0.89	1.42 ± 0.93	1.33 ± 1.02	1.58 ± 0.99	2.178	0.143	0.019	5.986	0.016	0.050	0.002	0.966	0.000
Duration_PSQI	2.07 ± 0.86	1.64 ± 0.10	1.12 ± 0.83	1.64 ± 1.10	0.087	0.769	0.001	37.844	0.000	0.289	43.088	0.000	0.274
Efficiency_PSQI	1.58 ± 1.05	1.47 ± 1.04	1.53 ± 0.97	1.54 ± 1.12	0.058	0.811	0.001	0.014	0.905	0.000	0.912	0.342	0.008
Discomfort_PSQI	2.35 ± 0.55	2.19 ± 0.78	1.16 ± 0.68	1.95 ± 0.96	6.865	0.010	0.0.57	89.484	0.000	0.440	39.953	0.000	0.260
Medication use_PSQI	1.67 ± 0.76	1.83 ± 0.95	1.95 ± 0.88	1.56 ± 1.06	0.666	0.416	0.006	0.002	0.963	0.000	7.447	0.007	0.061
Dysfunctions during the day_PSQI	0.72 ± 0.70	0.69 ± 0.77	0.65 ± 0.69	0.46 ± 0.54	0.901	0.344	0.008	7.734	0.006	0.064	2.285	0.133	0.020
Total score_PSQI	10.77 ± 2.98	10.07 ± 2.90	8.88 ± 2.53	10.31 ± 3.26	0.507	0.478	0.004	17.942	0.000	0.136	29.687	0.000	0.207

Data are expressed as the mean and standard deviation. Qualitative variables are presented as frequency and percentage. CG = control group; EG = experimental group; HADS = Hospital Anxiety and Depression Scale; PSQI = Pittsburgh Sleep Quality Scale; PSS = Perceived Stress Scale.

## Data Availability

The data presented in this study are available upon request from the corresponding author. The data are not publicly available because, due to the sensitive nature of the questions asked in this study, participants were assured raw data would remain confidential and would not be shared.
